# Invariant Domain Watermarking Using Heaviside Function of Order Alpha and Fractional Gaussian Field

**DOI:** 10.1371/journal.pone.0123427

**Published:** 2015-04-17

**Authors:** Almas Abbasi, Chaw Seng Woo, Rabha Waell Ibrahim, Saeed Islam

**Affiliations:** 1 Department of Artificial Intelligence, Faculty of Computer Science and Information Technology, University of Malaya, Kuala Lumpur, Malaysia; 2 Institute of Mathematical Science, University of Malaya, Kuala Lumpur, Malaysia; 3 Department of Mathematics, Abdul Wali Khan University, Shankar, Pakistan; Glasgow University, UNITED KINGDOM

## Abstract

Digital image watermarking is an important technique for the authentication of multimedia content and copyright protection. Conventional digital image watermarking techniques are often vulnerable to geometric distortions such as Rotation, Scaling, and Translation (RST). These distortions desynchronize the watermark information embedded in an image and thus disable watermark detection. To solve this problem, we propose an RST invariant domain watermarking technique based on fractional calculus. We have constructed a domain using Heaviside function of order alpha (HFOA). The HFOA models the signal as a polynomial for watermark embedding. The watermark is embedded in all the coefficients of the image. We have also constructed a fractional variance formula using fractional Gaussian field. A cross correlation method based on the fractional Gaussian field is used for watermark detection. Furthermore the proposed method enables blind watermark detection where the original image is not required during the watermark detection thereby making it more practical than non-blind watermarking techniques. Experimental results confirmed that the proposed technique has a high level of robustness.

## 1. Introduction

The watermarking techniques are useful for purpose of content authentication and copyright protection. Digital image watermarking inserts information called watermark in an image by modifying image pixels values in such a way that the difference between the original and watermarked images is hardly noticeable.

Digital watermarking can be used in different areas which require content authentication and copy right protection. To protect the copyrights the watermark should be imperceptible and robust against variety of attack. It includes robustness against noise addition attack, JPEG Compression attack, filtering, and other common image processing attacks. The basic requirement of a Watermarking technique is to achieve high imperceptibility and robustness.

Watermarking methods can be categorized into two types, spatial domain [1–3] and transform domain [4–6]. In spatial domain methods the watermark is embedded directly into the pixels of the original image.

Least significant bit (LSB) embedding is an example of spatial domain method. The watermark embedded in the least bit is least perceive by human eye. However the spatial domain methods are not robust i-e. They cannot survive various kinds of attacks as the least bit are usually removed or destroyed in these attacks.

Transform domain methods embed the watermark into the transformed coefficients of the original image. Transform based methods are most popular as they are generally more robust to malicious attacks. In transform domain method the transformed coefficient of the original image is altered to embed watermark. Researchers have considered many image transformations. Most popular transform domains are discrete cosine transformation (DCT), discrete wavelet transforms (DWT), and discrete Fourier transforms (DFT).

Researchers have also used polynomial based transformation for watermarking. The author in [7] proposed chirp watermark detection using discrete polynomial phase transform (DPT). The DPT models the signal as polynomial. Watermark is represented by the phase of DPT. They found DPT detect watermark with high accuracy. The author proposed an image adaptive invisible watermark based on orthogonal polynomial transformation (OPT) in [8]. They generate Just Noticeable Distortion mask (JND) by taking into account the image features such as textures, edges, and luminance of the cover image in the OPT domain to embed the watermark imperceptibly.

Moment domain based watermarking techniques [9] has been proposed by scientists. [10] Proposed a Moment domain based watermarking algorithm. The proposed invariant watermarking technique used image features for watermark embedding. They use Zernike moment of the image to obtain Rotation, Scale and Translation invariance.

Most of abovementioned methods embed watermark in specific area called feature points usually located in patches. Exhaustive search is performed to determine features points for watermark embedding, which is time consuming task. Similarly in watermark detection phase, again search operation is performed for correlation operation. Such methods are also very sensitive to cropping attack where a portion of watermarked image is removed. In such situation watermark detection becomes impossible.

In our proposed technique we achieve an invariant domain using Heaviside function of order alpha. Watermark is embedded in all the coefficients. Moreover we have also constructed cross correlation method based on fractional Gaussian field for watermark detection.

The rest of the paper is structured as follows. Section 2 covers the construction of our proposed technique details, including watermark embedding and detection process. In Section 3 experimental results are presented. Conclusions are drawn in Section 4.

## 2. Proposed Technique

The watermarking domain used in this technique is transform domain based on HFOA. The watermark embedding and extraction is performed in HFOA. Further the watermarking technique used in this study is blind watermarking technique. In blind watermarking technique original image is not required for the watermark detection and is more practical than non-blind watermarking techniques.

### 2.1 Fractional calculus

Fractional calculus and its applications (that is the theory of derivatives and integrals of any arbitrary real or complex order) has importance in several widely diverse areas of mathematical physical, computer sciences and engineering sciences. It generalizes the ideas of integer order differentiation and n-fold integration. Fractional derivatives are an excellent instrument for the description of general properties of various materials and processes such as signal processing and image processing. Recently researchers has successfully applied fractional calculus operators (differential and integral) for enhancing image quality, edge detection and image restoration operations etc [11–15]. One of these famous operators is the Riemann-Liouville operator (differential and integral), which defined by

**Definition 2.1.** The fractional (arbitrary) order integral of the function of order α>0 is defined by

$$I_{a}^{\alpha }f(t)=\int_{a}^{t}\frac{{\left(t-\tau \right)}^{\alpha -1}}{\Gamma (\alpha )}f(\tau )d\tau$$

**Definition 2.2.** The fractional (arbitrary) order derivative of the function of order 0<α<1 is defined by

$$D_{a}^{\alpha }f(t)=\frac{d}{dt}\int_{a}^{t}\frac{{\left(t-\tau \right)}^{-\alpha }}{\Gamma (1-\alpha )}f(\tau )d\tau =\frac{d}{dt}I_{a}^{1-\alpha }f(t).$$

**Remark 2.1.** From Definition 2.1 and Definition 2.2, we have

$$\begin{array}{l} D^{\alpha }t^{u}=\frac{\Gamma (\mu +1)}{\Gamma (\mu -\alpha +1)}t^{\mu -\alpha },\mu >-1;0<\alpha <1\\ I^{\alpha }t^{\mu }=\frac{\Gamma (\mu +1)}{\Gamma (\mu +\alpha +1)}t^{\mu +\alpha },\mu >-1;\alpha >0. \end{array}$$

In our investigation, we utilize the Heaviside basis functions

**Definition 2.3**. By utilizing the Leibniz rule between two functions *φ* and *f* We have

Datp(ϕ(t)f(t))=∑k=0∞(pk)ϕ(k)(t)Datp−kf(t)

By substituting

f(t)=H(t−α) 1

where *H(t–α)* the Heaviside is function of α.

Dαtp(ϕ(t)f(t))=∑k=0∞(pk)ϕ(k)(t)Dαtp−kH(t−α)H(t−α)                                =(t−α)−pΓ(1−p)ϕ(k)(t)Dαtp−kH(t−α)                               =(t−α)−pΓ(1−p)ϕ(t)+∑k=1∞(pk)(t−α)k−pΓ(k−p+1)ϕ(k)(t)                              =∑k=0∞(pk)(t−α)k−pΓ(k−p+1)ϕ(k)(t)

Where $\left(\begin{array}{l} p\\ k \end{array}\right)=\frac{p!}{k!\left(p-k\right)!}$ and *φ* ^*(k)*^is the *K*orientation of the function *φ*(t)and Γ is the Euler gamma function.

### 2.2 Properties of Heaviside

#### 1. Linearity:

$$H(ax+b)=\left\{ \begin{array}{l} H\left(x+\frac{b}{a}\right)a>0\\ H\left(-x-\frac{b}{a}\right)a<0 \end{array}\right.$$

#### 2. Invariance property:

a. Translation

$$H(x-x_{o})=H(•-x_{o})(x)$$

b. Scaling

Let $a=\frac{1}{c},c\neq 0,a>0$

$$H\left(\frac{x}{c}\right)=H\left(\frac{•}{c}\right)(x)$$

#### 3. Self-reversibility:

H(x)∗H(x)=tH(t)

### 2.3 Watermark embedding

Let *I* (*i*, *j*) represent the original gray scale image of size *M*×*N* pixels and *w* (*i*, *j*) is the watermark pattern to be embedded using additive embedding technique. Generally, additive embedding is implemented by using *I’ = I+ k w*, where *I’* is the watermarked image, and *k* is the embedding strength. The size of the watermark *w* is equal to the size of the image selected for watermark embedding. The strength of watermark *k* is kept as a constant value.

**Fig 1:**
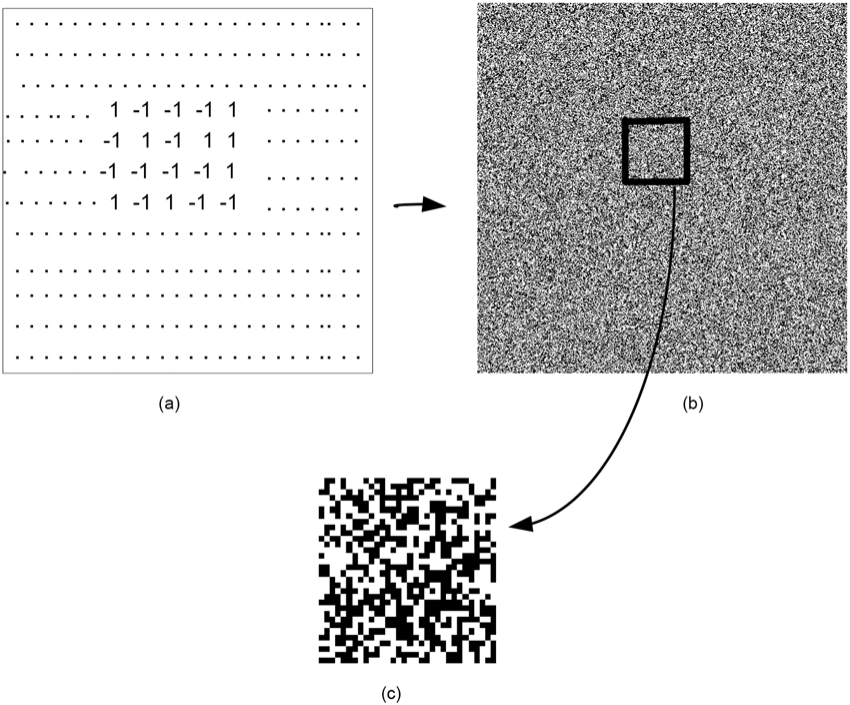
(a)Watermark consist of random sequence of +1, -1. (b-c) Watermark pattern.

**Fig 2:**
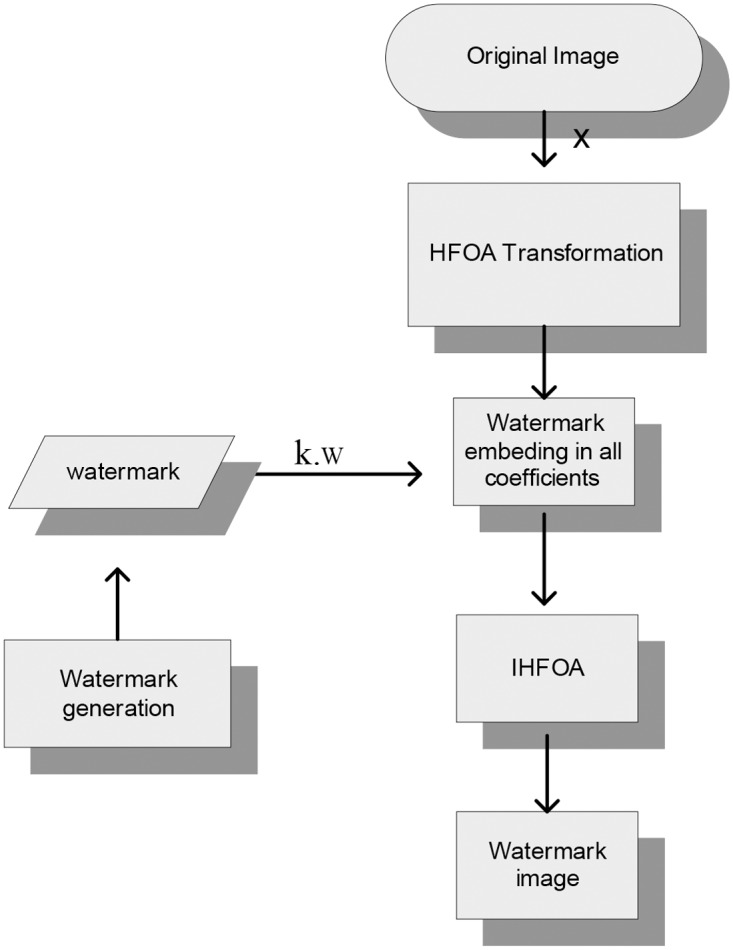
Watermark embedding scheme for the Invariant domain.

For the current implementation, the watermark signal consists of {+1,-1} bits as shown in Fig 1. The watermark embedding process can be represented by the following equation and is shown in Fig 2:

$$S=s+(w_{b}\times \kappa )$$ 2

where *S* represents the watermark signal, *s* is the original signal (i.e. coefficients in our case), *w* ^*b*^ is the watermark bit. The parameter κ is a constant value.

The proposed watermarking embedding technique includes the following steps:

- Read the grayscale images of size 512×512.
- Apply fractional H_α_ transformation:
  - Set the parameterα,0<α≤1.
  - Fix the value of the variable *t* such that t>α
  - Calculate the fractional order of H_α_ polynomial using Definition 2.3
- Embed the watermark using the Eq (2).
- Calculate inverse fractional Hα by taking transpose of the resultant image.
- Perform steps i to iv for each image.

### 2.4 Watermark detection

**Fig 3:**
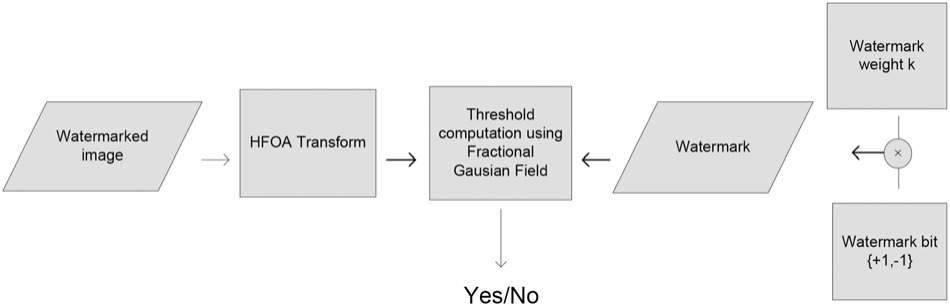
Watermark Detection.

Watermark detection is the reverse of the embedding processes and it is shown in Fig 3. Our aim is to extract the watermark using fractional Gaussian field [6]. The fractional Gaussian field has a simple covariance structure and it is related to two generalizations of fractional motion known as multifunction motions. The Gaussian field due to its inherent duality reveals a new way of constructing martingales associated with the odd and even parts of a fractional motion.

Assume the image I_α_ of size MN, where the M is the number of rows and N is the number of columns. Then the covariance is given by [6] which can be represented as follows:

$$E(I_{\alpha i}(M),I_{\beta j}(N))=A_{\alpha ,\beta }*a$$ 3

Where *α* can be represented as:

$$a=\left(\frac{M^{\alpha +\beta }+N^{\alpha +\beta }-{\left|M-N^{}\right|}^{\alpha +\beta }}{2}\right)$$ 4

$$E(I_{\alpha i}(M),I_{\beta j}(N))=A_{\alpha ,\beta }\left(\frac{M^{\alpha +\beta }+N^{\alpha +\beta }-{\left|M-N^{}\right|}^{\alpha +\beta }}{2}\right)$$ 5

Where α, β are fractional numbers. In practice the following unbiased estimate of *E(I*_*αi*_,*I*_*βj*_ *)* is used

$$\begin{array}{l} E(I_{\alpha i},I_{\beta j})\approx (I_{\alpha i},I_{\beta j})=A_{\alpha +\beta }\left(\frac{M^{\alpha +\beta }+N^{\alpha +\beta }-{\left|M-N^{}\right|}^{\alpha +\beta }}{2}\right) \end{array}$$ 6

where *A*_*αi+βj*_ can be represented by the following equation:

$$\begin{array}{l} A_{\alpha i+\beta j}=-\frac{2}{\pi }\left(\sqrt{\Gamma \left(2\alpha +1\right)\sin\left(\alpha \pi \right)\Gamma \left(2\beta +1\right)\sin\left(\beta \pi \right)}\right)\times \\ \Gamma \left(-\left(\alpha +\beta \right)\right)\cos\left(\left(\beta -\alpha \right)\frac{\pi }{2}\right)\cos\left(\left(\alpha +\beta \right)\frac{\pi }{2}\right) \end{array}$$ 7

for *α+β*≠ 1 and

$$A_{\alpha }=\sqrt{\Gamma (2\alpha +1)\Gamma (3-2\alpha })\sin^{2}(\alpha \pi )$$ 8

for *α+β* = 1.

In general the variance of population of size *N*, is represented as:

$$\begin{array}{l} \sigma_{}^{2}=\frac{1}{N}{\sum_{i=1}^{n}\left(E\left[X\right]\right)}^{2}\\ \sigma_{}^{2}=\frac{1}{MN}{\sum_{i=0}^{M-1}\sum_{j=0}^{N-1}\left(E{\left(I_{\alpha i},I_{\beta j}\right)}^{}\right)}^{2} \end{array}$$

In practice the following unbiased estimate of σ^2^ *αβ* is used:

$$\sigma_{\alpha \beta }^{2}\approx \frac{1}{MN}{\sum_{i=0}^{M-1}\sum_{j=0}^{N-1}\left[(A_{\alpha ,\beta }*a)I^{'}\left(i,j\right)\right]}^{2}$$ 9

where *I*΄(*i*,*j*) is the watermark coefficients and (A_*α*,β_*α) is defined in Eq (3).

The correlation between the marked coefficients and the watermarked sequence to be tested for the presence is computed as:

$$\rho =\frac{1}{MN}\sum_{i=0}^{M-1}\sum_{j=0}^{N-1}\left[\left(A_{\alpha ,\beta }*a)I^{'}\left(i,j\right)w(i,j\right)\right]$$ 10

we used the following threshold:

$$T_{\rho }=3.97\sqrt{2\sigma_{\alpha \beta }^{2}}$$ 11

The proposed technique watermark extraction steps are summarize in the following pseudo code:

- Read the watermark/attacked image.
- Apply HFOA transformation to the image.
- Compute *ρ* and *T*_*ρ*_ using Eqs (10) and (11) respectively.
- Compare *ρ* and *T*_*ρ*_, If *ρ* > *T*_*ρ*_ then watermark is detected otherwise not.
- Repeat step i to iv for all images.

## 3. Experiments Analysis

In this section, we evaluated the performance of the proposed watermarking technique by considering robustness and imperceptibility. Five test images, namely, Bridge, House, Sunset, Building, and Boat, were considered for evaluation purposes. Each of these images were of size 512×512 pixels and they are shown in Figs A-E in S1 File. As a proof of concept, the algorithm was coded by using Matlab and checkmark software [16] was deployed for testing the robustness against different set of attacks. The watermark signal presented by the sequence of +1 or -1.

### 3.1 Imperceptibility

The watermarked images obtained by using the proposed technique are shown in Figs F-J in S1 File. In each image 262144 bits of watermark were embedded. By visual inspection, the watermarked images appear perceptually similar to their original counterparts. To quantify the transparency of the embedded watermark, the Peak signal to noise ratio (PSNR) and Structure similarity (SSIM) index were considered. These are popular metrics employed by the watermark community. SSIM index defines a way to measure the similarity between two images. The SSIM index was computed locally using 11x11 circular Gaussian sliding window. In order to measure similarity, SSIM index take into account the similarity of luminance, similarity of contrast and similarity of structure of the two compared images i-e original image and watermarked image. These similarities were calculated by mean of simple statistics. The luminance similarity were calculated by using local mean of two images. Similarly contrast similarity was calculated using local standard deviation while cross correlation after removing the mean value was used to calculate structure similarity between the two images. The resulting values were combined using exponents alpha, beta, and gamma, averaged to produce a final SSIM index value as calculated in [17]. The results are recorded in Table 1. It was observed that the PSNR and SSIM values range from 35 to 38 dB and 0.76 to 0.93, respectively. These readings suggest that the watermark image generated by the proposed method is of good perceptual quality.

**Table 1:** PSNR and SSIM value of sample test images in the proposed HFOA domain.

	Boat	Bridge	Sunset	House	Building
**Peak signal to noise ratio (dB)**	37	35	38	37	36
**Mean square error**	12	19	14	14	14
S**tructure similarity index measure**	0.85	0.93	0.76	0.86	0.86

### 3.2 Robustness

The watermarked images had undergone various types of attack to investigate the robustness of the proposed technique. In particular, each watermarked image was distorted using different geometric and image processing attacks, namely: (1) scaling attack with scaling factor ranging from 0.5 to 2; (2) JPEG compression with quality factor ranging from 10% to 90% with increment of 10; (3) row and column removal attack with varying rows and column removal from 1 to 17; (4) Gaussian filtering with kernel size of 3×3 and 4×4 pixels; (5) Cropping attack with cropping area of different size such as 25 × 26 pixels, 54 × 54 pixels and 149 × 149 pixels; (6) Rotation attack with cropping option, having rotation angle from 5 to 90 degrees; (7) Circular shift attack 50% of original image size 512×512 pixels.

**Fig 4:**
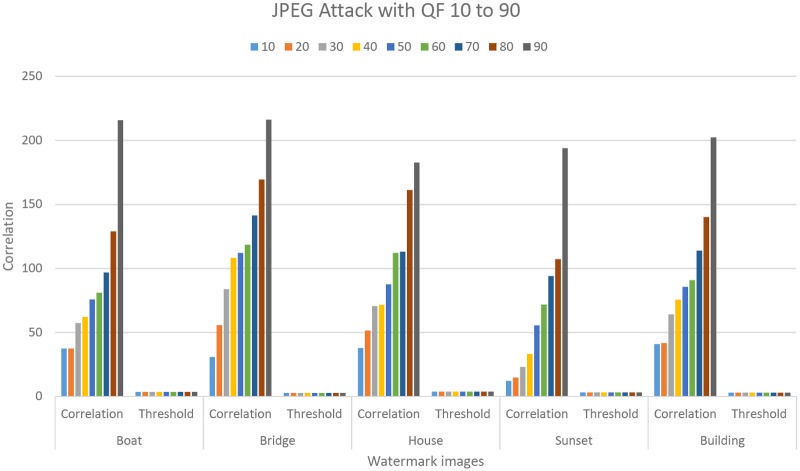
Robustness against JPEG compression attack. Five watermarked images are tested for the JPEG compression quality factor ranging from 10 to 90.

**Fig 5:**
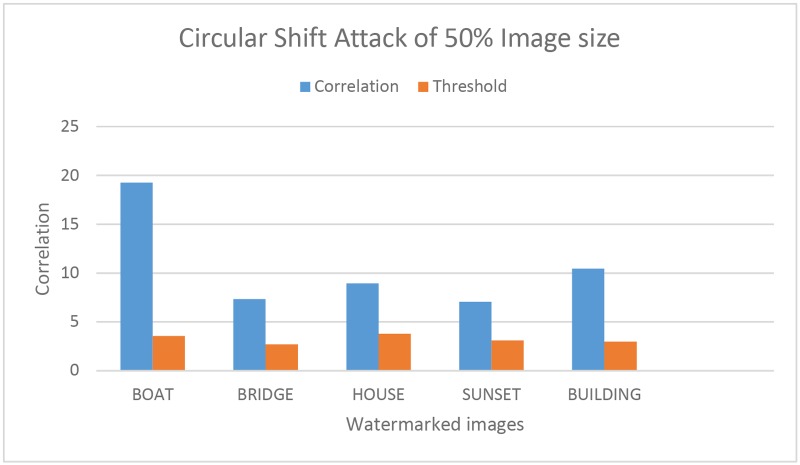
Robustness against circular shift Attack. Comparison of the Correlation and Threshold values of five watermarked test images against the circular shift attack.

**Fig 6:**
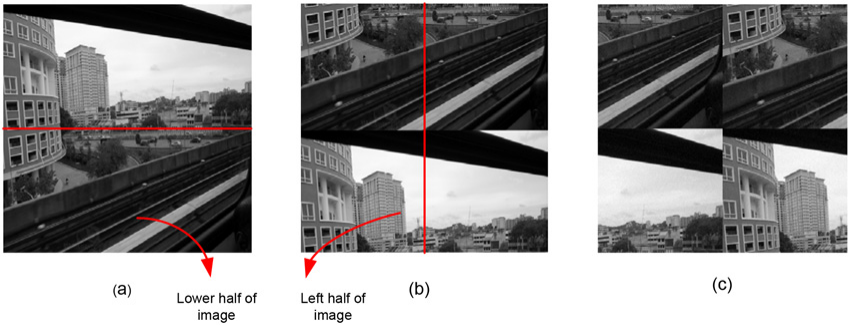
Circular shift attack operation of 50% image size. (a) Original image. (b) Pixels at lower half of the image shifted upwards. (c) Circular shift attacked image. Pixels at left half of image shifted towards right hand side of the image.

**Fig 7:**
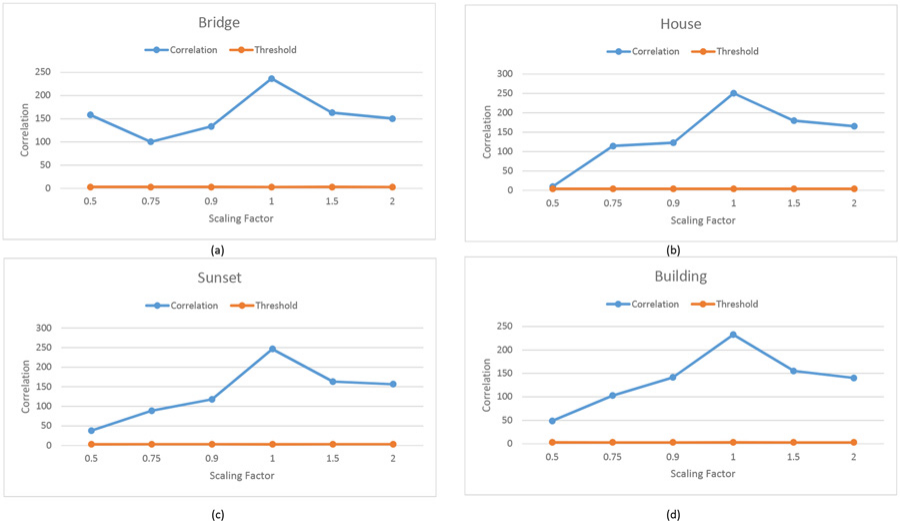
Robustness against scaling Attack. (a-d) Comparison of Correlation values of four images after scaling attack of the proposed technique.

**Fig 8:**
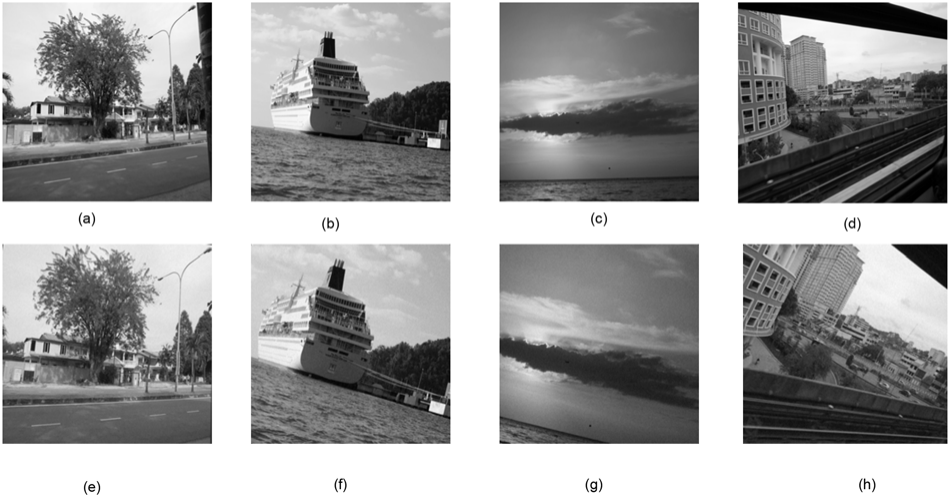
Robustness against rotation attack. The rotated images have also been scaled and cropped. (a-d) Original Test Images, (e-h) Attacked watermarked Test Images: Rotation attack for different values of the angles are taken such as 5, 10, 15 and 30 degree respectively.

**Fig 9:**
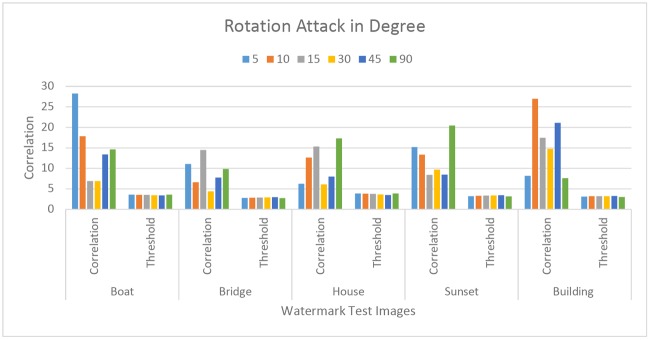
Robustness of Proposed Technique against Rotation attacks. Five watermarked images are tested for the rotation attack. Rotation angle is taken of different values such as 5, 10, 15, 30, 45 and 90 degrees.

**Fig 10:**
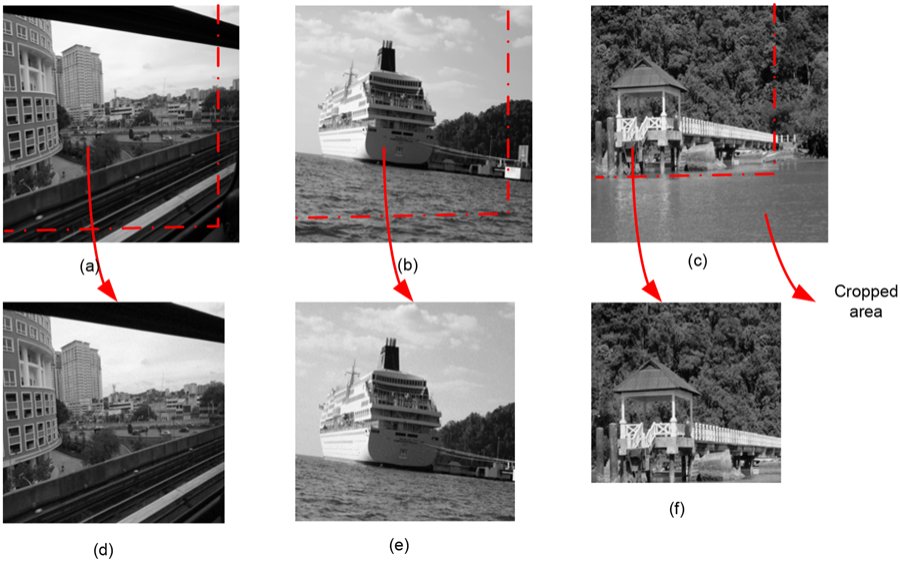
Robustness of Proposed Technique against Cropping attack. (a-c) Original images of size 512×512 pixels. (d) strips 25 and 26 pixels wide were cropped from the bottom and right hand side of the image respectively. The size of the image after cropping attack is 487×486 pixels. (e) strips 54 pixels wide were cropped from the bottom and right hand side of the image. The size of the image after cropping attack is 458×458 pixels. (f) strips 149 pixels wide were cropped from the bottom and right hand side of the image. The size of the image after cropping attack is 363×363 pixels.

Robustness of the proposed technique against JPEG compression attack with quality factor ranging from 10 to 90 were tested. Fig 4 represent the comparison of correlation value against the threshold value, after the JPEG attack. The result confirms that the proposed technique is robust against the JPEG compression attack. Further we evaluated the performance of the proposed technique under different attacks and the results are summarized in Figs 5–10. The cross correlation computation based on fractional Gaussian field criterion *ρ* and the threshold value *T*_*ρ*_ are considered to test the presence of the embedded watermark.

Robustness against RST attack had been tested successfully. Fig 5, represents the correlation value of five watermark images after the circular shift attack. The correlation value of the attack image remains above the threshold value which confirm that the proposed technique is robust against the circular shift attack. The circular shift operation is set as the 50% of the image size. It circularly shift the image pixels rows and columns. The circular shift operation has been described in Fig 6. Fig 7a–7d), represent the correlation values of four watermark images after scaling attack using scaling factors ranging from 0.5 to 2.0, *ρ* remains well above the *T*_*ρ*_ (i.e., threshold). Therefore, the results suggest that the proposed method is robust against the scaling attack. Last but not the least Figs 8 and 9, represents the performance of the proposed technique against rotation attack. Fig 9 represent the comparison of correlation value against the threshold value, after the rotation attack. The result confirms that the proposed technique is robust against the rotation attack. Fig 10, shows image after performing the cropping attack with cropping area of different size such as 25 × 26 pixels, 54 × 54 pixels and 149 × 149 pixels. The result shows high level of robustness of our proposed technique as the watermark has been detected in all the images.

### 3.3 Comparison with other techniques

In this section, we compare the performance of the proposed watermarking technique by considering robustness. Five standard test images from the USC-SIPI dataset, namely, Baboon, Cameraman, Lena, Peppers, and Sailboat, are considered for comparison purposes. These images are each of dimensions 512×512. The algorithm is coded by using Matlab and checkmark software [16] is deployed for testing the robustness against different set of attacks.

A direct comparison with similar work is made here involving Kim et al.’s method [10]. A value “1” in the watermark detection column of Table 2 indicates that watermark is detected in all images for the specified category of attack, while a “x” indicates no simulation result has been reported in the referenced literature. The experimental results recorded in Table 2 confirms that our proposed technique has good level of robustness while maintaining good imperceptibility.

**Table 2:** Comparison of robustness test with other technique.

Attack category	Description	Watermark Detection	
	Description of attacks	Technique[10]	Proposed Technique
Rotation with cropping option	Angle in degrees: -2 to 45	1	1
Scaling	0.5 to 2.0	0.870	1
Circular shift	50% of image size(512x512)	1	1
Cropping	50% of image size(512x512)	1	1
JPEG Compression	10% to 90%	0.900	1
Random Bending	Random Bending attack	0.950	1
Row and column removal	Remove 1 to 17 rows and columns	x	1
Aspect ratio change	Change aspect ratio in x and y direction	x	1
Gaussian Filtering	Kernel size 3x3,4x4	x	1
Shearing	Shearing in x and y direction	x	1
Sharpening	Unsharp filter	x	1

For scaling attack, our technique achieved 100% detection while, [10] yield 87% detection. For circular shift attack, no result was reported in [10], but the proposed method achieve 100% detection.

For the JPEG compression and random bending attacks, Kim et al.’s method yields 90% and 95% watermark detection respectively, while the proposed method achieve 100% watermark detection for both attacks. For the other types of geometric attack such as row column removal attack, aspect ratio attack, Gaussian filtering attack, sharpening and shearing, proposed method achieve 100% detection, but the aforementioned attacks are not considered in Kim et al.’s method.

With the aforementioned observations, we conclude that the proposed method performs better than the conventional RST invariant watermarking methods considered.

## 4. Conclusion

Digital image watermarking is an important technique for the authentication of multimedia content and copyright protection. We introduce invariant watermarking algorithm based on the fractional calculus. We have constructed a domain using HFOA. The HFOA model the signal as a fractional polynomial for watermark embedding. We have also constructed cross correlation method based on the fractional Gaussian field for watermark detection. Experimental results confirmed that the proposed technique is highly robust against image processing and geometric attacks.

## Supporting Information

S1 File Original and watermark Test Images. (ZIP)
