# Supplementary figures and images for: Invariant Domain Watermarking Using Heaviside Function of Order Alpha and Fractional Gaussian Field
Source: PLoS One. 2015 Apr 17;10(4):e0123427. doi: 10.1371/journal.pone.0123427 (PMC4401616; doi:10.1371/journal.pone.0123427)

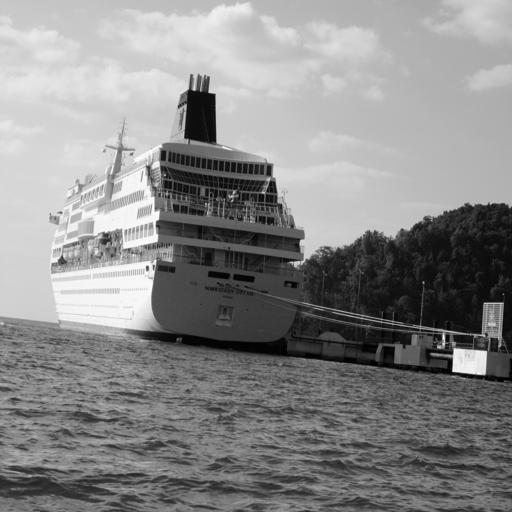

Supplement: S1 File — (ZIP) [file pone.0123427.s001.zip › supporting_information/FigureA.tif]

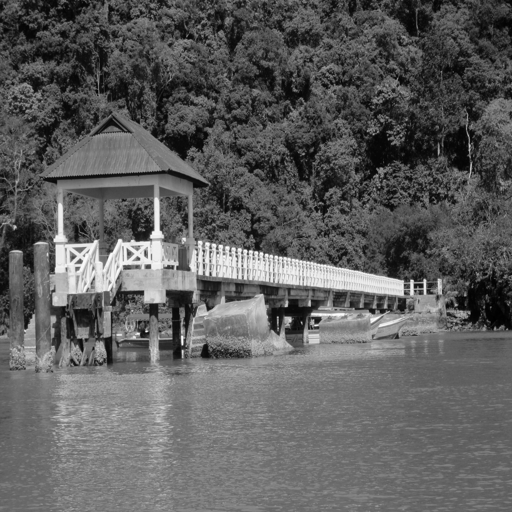

Supplement: S1 File — (ZIP) [file pone.0123427.s001.zip › supporting_information/FigureB.tif]

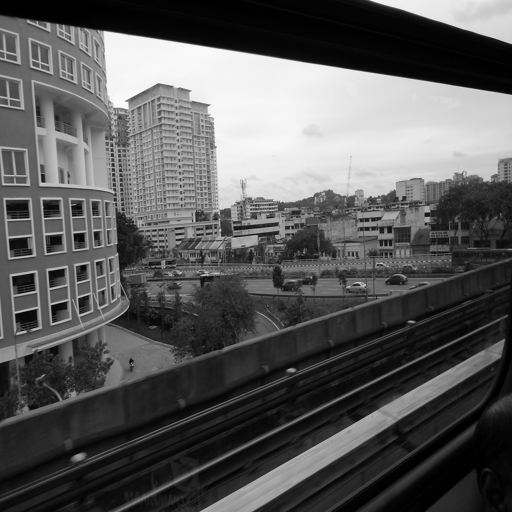

Supplement: S1 File — (ZIP) [file pone.0123427.s001.zip › supporting_information/FigureC.tif]

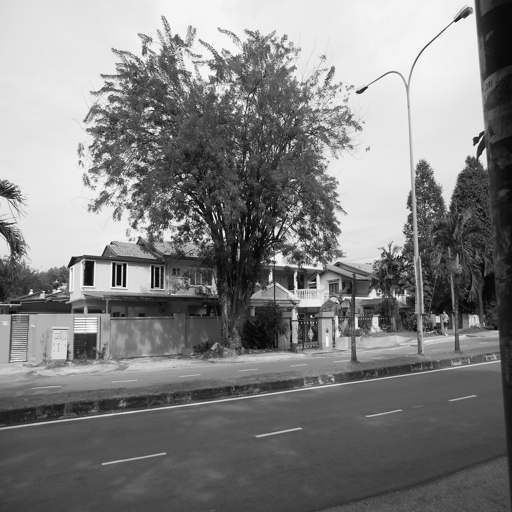

Supplement: S1 File — (ZIP) [file pone.0123427.s001.zip › supporting_information/FigureD.tif]

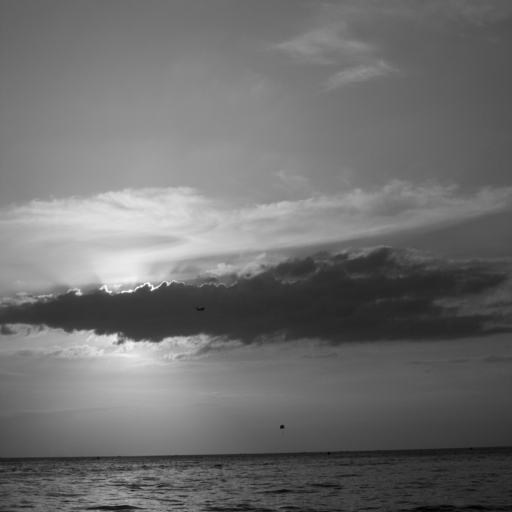

Supplement: S1 File — (ZIP) [file pone.0123427.s001.zip › supporting_information/FigureE.tif]

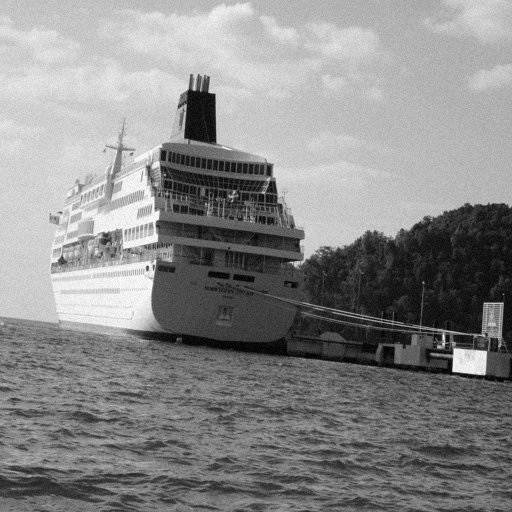

Supplement: S1 File — (ZIP) [file pone.0123427.s001.zip › supporting_information/FigureF.tif]

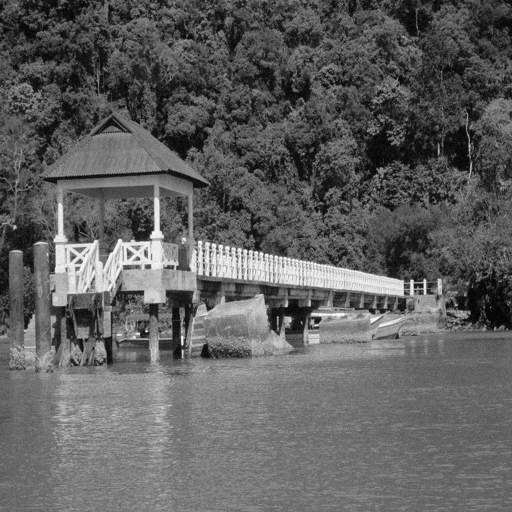

Supplement: S1 File — (ZIP) [file pone.0123427.s001.zip › supporting_information/FigureG.tif]

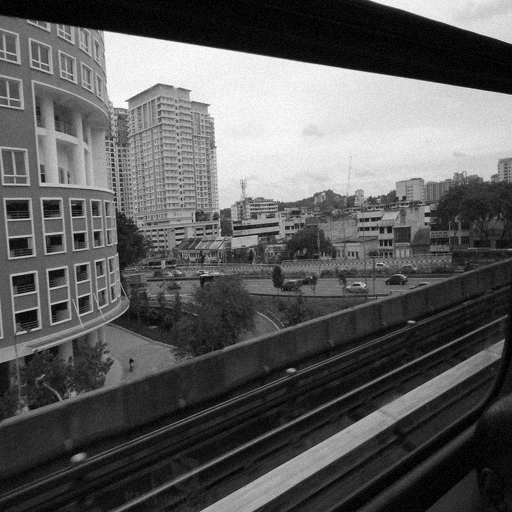

Supplement: S1 File — (ZIP) [file pone.0123427.s001.zip › supporting_information/FigureH.tif]

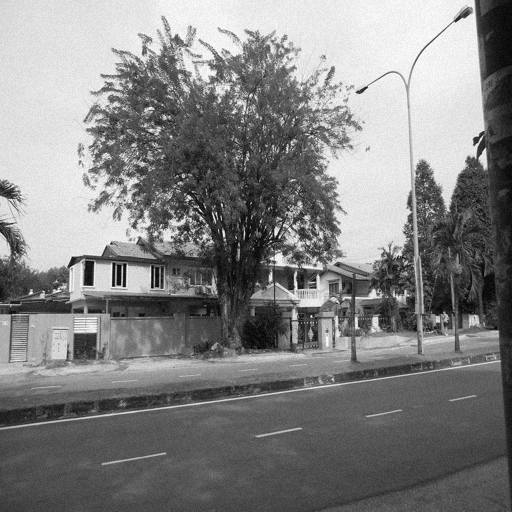

Supplement: S1 File — (ZIP) [file pone.0123427.s001.zip › supporting_information/FigureI.tif]

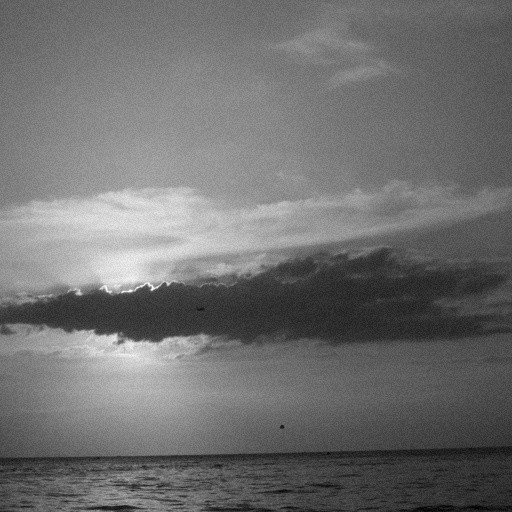

Supplement: S1 File — (ZIP) [file pone.0123427.s001.zip › supporting_information/FigureJ.tif]
